# Recent Advances in Copper-Doped Titanium Implants

**DOI:** 10.3390/ma15072342

**Published:** 2022-03-22

**Authors:** Yuncheng Wu, Hao Zhou, Ye Zeng, Hongxing Xie, Dongxu Ma, Zhoucheng Wang, Hanfeng Liang

**Affiliations:** Department of Chemical and Biochemical Engineering, College of Chemistry and Chemical Engineering, Xiamen University, Xiamen 361005, China; wuyuncheng@stu.xmu.edu.cn (Y.W.); 20420201151864@stu.xmu.edu.cn (H.Z.); 20620190154094@stu.xmu.edu.cn (Y.Z.); 15810878679@163.com (H.X.); ma_dx@163.com (D.M.)

**Keywords:** implant, titanium and titanium alloys, copper doping, synthetic methods, implant–bacteria interactions, biocompatibility

## Abstract

Titanium (Ti) and its alloys have been extensively used as implant materials in clinical practice due to their high corrosion resistance, light weight and excellent biocompatibility. However, the insufficient intrinsic osteogenic capacity of Ti and its alloys impedes bone repair and regeneration, and implant-related infection or inflammation remains the leading cause of implant failure. Bacterial infections or inflammatory diseases constitute severe threats to human health. The physicochemical properties of the material are critical to the success of clinical procedures, and the doping of Cu into Ti implants has been confirmed to be capable of enhancing the bone repair/regeneration, angiogenesis and antibacterial capability. This review outlines the recent advances in the design and preparation of Cu-doped Ti and Ti alloy implants, with a special focus on various methods, including plasma immersion implantation, magnetron sputtering, galvanic deposition, microarc oxidation and sol-gel synthesis. More importantly, the antibacterial and mechanical properties as well as the corrosion resistance and biocompatibility of Cu-doped Ti implants from different methods are systematically reviewed, and their prospects and limitations are also discussed.

## 1. Introduction

Titanium (Ti) and its alloys have been widely used in human implants, such as dental implants, hip and knee replacements, bone plates and screws, owing to their good mechanical properties, excellent corrosion resistance and optimal biocompatibility [1,2,3]. However, these materials are susceptible to bacterial infections because of their biological inertness, low osteogenic and insufficient antibacterial ability [4,5]. In orthopedic surgery, periprosthetic infections can lead to the loosening of the prosthesis, failure of joint fusion, amputation and even death.

For dental implants, inflammatory disease around the implants often leads to the loss of surrounding bones and consequently affects the longevity of the implants, which is the most common cause of implant failure. The average prevalence of peri-implantitis was 22%, with a positive correlation to the time of placement [6], whereas 5–11% of implants were failed and must be removed [7]. For orthopedic replacement, the risk of infection is around 0.4% to 16.1%, depending on the extent of fracture [8,9]. After total knee arthroplasty, the probabilities of periprosthetic joint infection at the knee, hip and ankle locations were 0.5–2% [10], 2–9% [11] and 0.3–1.7% [12], respectively.

Moreover, in dentistry, the inflammatory response to peri-implantitis was as high as 6.47% during the five years observation period and from 5.8% to 16.9% after more than ten years of implantation [13]. These postoperative complications and the lack of effective treatments can significantly increase hospital stay and costs, leaving the patient in a stressful, distressed and critical condition. Therefore, preventing bacterial invasion is essential to ensure the success of implantation, and improving the antimicrobial properties of implant materials is a pressing need.

An optimal biomaterial surface with antimicrobial properties will prevent bacterial adhesion during the initial stages of infection and inhibit subsequent bacterial growth at a later stage. At the same time, the implant is considered as a foreign body during osseointegration, and the formation of new primary bones accompanied by remodeling of healthy bones also plays a key role in the long-term stability of the implants. Angiogenesis is important for bone regeneration, as the formation of new blood contributes to nutrient delivery and promotes bone repair, especially in wide area bone injuries.

Therefore, the clinical success depends not only on osseointegration but also on peri-implant neovascularization. In addition, a significant increase in antibiotic-resistant strains has been observed, calling for innovative prevention and treatment strategies [14]. Copper (Cu) is an important human trace element with low cytotoxicity and is an effective antibacterial additive. Thus, Cu has been widely used in biomedical materials to improve their antimicrobial capacity [2], which can prevent device-related infections without severe biosafety-related problems [15,16].

In recent years, many pieces of research have shown that Cu-doped Ti implants are resistant to bacterial adhesion and biofilms due to the release of Cu ions from the coating surface. In addition to the antibacterial effects, Cu ions could also enhance the differentiation of bone marrow stem cells (BMSCs) to osteoblasts, which is associated with bone regeneration [17]. Therefore, introducing Cu can give Ti and its alloy implants improved osteogenesis ability without reducing the biocompatibility.

In this paper, we aim to provide a timely review on recent progress in Cu-doped Ti implants, covering the preparation technology, mechanical properties as well as the antimicrobial and bone-enabling behaviors. We first describe the bacterial infection and osseointegration process of medical implants and then summarize the recent advances in the surface modification of Ti-based medical implants with Cu dopants. Last, we discuss the existing issues and potential future directions.

This review, however, will not discuss the antimicrobial coatings for Ti implants or other antimicrobial metals or alloys. Readers who are interested in these topics are recommended to look at the information provided elsewhere [18,19,20]. We hope that this review will deepen the understanding of the effect of Cu dopants and further provide useful guidance on the rational design of Cu-doped Ti implants with desirable properties.

## 2. Implant–Bacteria Interactions

Biomaterial-associated infections (BAI) often lead to the formation of biofilms on the surface of biomaterials and have been recognized as a major cause of surgical implant failure [21,22,23]. Biofilms are a hallmark of extracellular polymeric matrix production [24] since many biofilm-forming bacteria produce various extracellular polymeric substances (EPS) [25]. Biofilm formation, which is usually the result of bacterial adhesion, growth and colony formation, can weaken the effect of antibiotics due to the diversity of bacterial species and the barrier effect of extracellular polysaccharides [25,26,27,28,29].

**Figure 1:**
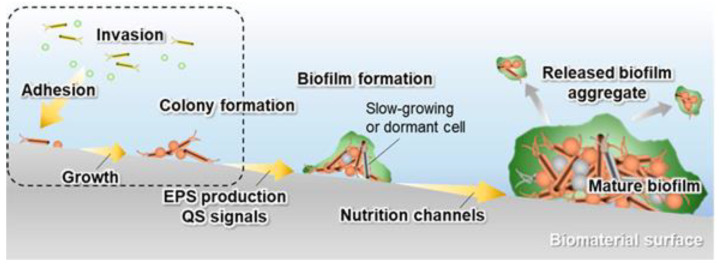
Schematic diagram of the biofilm formation process. The dashed area represents the initial stages of biofilm formation. Reproduced with permission from Ref. [30]. Copyright 2020, Multidisciplinary Digital Publishing Institute.

Moreover, because of the three-dimensional (3D) structure of biofilms and the physiological structure of adherent bacteria, biofilms are more resistant to various antimicrobial agents [26,27,28,29]. The establishment of biofilms is the end state of bacterial infection and persists despite treatment. Therefore, it is essential to prevent the formation of biofilms against biomaterial-associated infections. The mechanism of biofilm formation on the surface of biomaterials is shown in Figure 1 [30].

In order to develop antimicrobial coatings, it is essential to understand the interactions between bacteria and materials, which can be broadly divided into four steps:

1. The adhesion of bacteria to the surface of the material. This stage is heavily influenced by many variables, including the type of pathogen, the nature of the physiological fluid and the physicochemical properties of the material surface. Particularly, the roughness and surface topography have a strong influence at this stage [31]. The process of adhesion is reversible.
2. The bacterial colonization of the implant surface, which is mediated by specific molecular and cellular interactions [18]. In addition, bacteria aggregate and undergo irreversible attachment, which completely changes the chemical properties of the implant surface through their metabolites.
3. The biofilm maturation, microcolony formation and entrapment of planktonic bacteria in the extracellular polymeric substances (EPS). When bacteria form colonies on the surface, they produce exopolymer substances (mainly polysaccharides and other macromolecules), which contribute to biofilm formation. The biofilm can protect the bacteria from both fluid shear stress and the action of systemic pharmacological therapies [18].
4. The proliferation of bacteria under biofilm protection until the entire surface of the material is covered.

As mentioned above, biofilms protect bacteria from external damage. Therefore, the inhibition of bacterial adhesion and prevention of biofilm formation is the focus of current research. Since the biofilm formation is closely related to the interactions of bacteria and implant materials, a change of the surface property would essentially affect the formation process.

Therefore, surface modification has been regarded as an efficient strategy for suppressing the biofilm formation. Several studies have shown that Cu-doped Ti-based surfaces possess anti-adhesive properties that inhibit bacterial adhesion and can damage the surface of the attached bacteria [32,33,34]. It is worth mentioning that the Cu-containing metal alloys also exhibit similar functionality due to the release of Cu ions under physiological conditions [15,35,36,37,38,39,40].

## 3. Preparation Techniques and Related Properties of Cu-Doped Ti Implants

In this section, we will systematically summarize and discuss the preparation techniques and their impacts on the structure and related properties of Cu-doped Ti implants.

### 3.1. Ion Implantation

**Figure 2:**
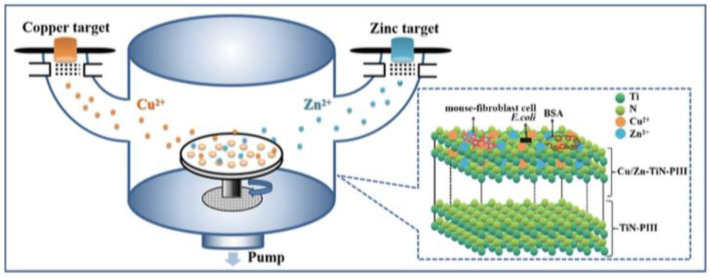
Schematic and structural diagrams of ion implantation. Reproduced with permission from Ref. [46]. Copyright 2019, American Vacuum Society.

Plasma immersion ion implantation (PIII) has been widely used for the modification of Ti surfaces. The PIII involves the injection of target metal ions as dopants into a suitable substrate utilizing pulsed, high-voltage direct current or pure direct current (Figure 2) [41,42], without changing the surface morphology of the substrate [19]. In addition, the technique also offers a high bond strength between the modified layer and the bulk material.

Compared to other methods, the PIII is highly reliable and reproducible [43] and is able to dope various elements, including Ag [43], Ca [44], Mg [45] and Zn [46] into Ti implants. More importantly, the amount of the dopants can be easily controlled by adjusting the plasma time, pressure, pulse duration and pulse frequency during the injection process [47], which provides a convenient and feasible way to tune the surface property.

It is now well established from various studies that the implantation of Cu ions into Ti by PIII technology has many advantages. Liu et al. [48] used the PIII technique to dope Cu into Ti implants and studied the effects of the Cu content on the bacterial and cellular behavior of Ti surface by adjusting the implantation time. They found that a shorter plasma treatment time (e.g., 1 h) resulted in a low Cu concentration and thus no significant antimicrobial effect on Escherichia coli (*E. coli*), while a longer plasma time (e.g., 2 or 3 h) led to an enhanced antimicrobial performance.

This is because, at a low Cu concentration, the bacteria were able to reach an intracellular Cu equilibrium; whereas, at a high Cu concentration, the bacteria can no longer maintain the intracellular Cu equilibrium, thus, making it impossible for bacteria to grow on the sample surfaces. Although the Cu ion release from Ti implants from 2 and 3 h plasma treatment was only slightly higher than that from the one made by 1 h treatment, the cell growth behavior on these samples were significantly different.

The former significantly facilitated the proliferation of HUVECs but barely affected the proliferation of rBMSCs, whereas the latter greatly promoted the proliferation of rBMSCs after 4 and 7 days of cell culture. This indicates that the HUVECs are more tolerant to Cu, while rBMSCs are more sensitive to Cu [48]. It is notable that the contact angle did not change significantly (approximately 100°) for samples injected with different copper contents, and thus the different responses exhibited by bacteria and cells to different samples are mainly due to the variation in copper content.

Guo et al. [49] illustrated that, with the increase of Cu content, Cu-doped calcium polyphosphate first promoted and then inhibited cell proliferation. It is speculated that the Cu-doped samples would become toxic to rBMSCs and HUVECs if a high Cu content is applied. Wan et al. [50] revealed that the implantation of Cu ions into stainless steel, pure Ti and Ti alloys significantly increased the mother samples’ antibacterial ability and wear resistance.

The corrosion resistance of 317 L decreased sharply after Cu ion implantation; while the corrosion resistance of Ti and Ti-Al-Nb decreased gradually as the ion dose increased. The ion implantation technique for the modification of biomaterials requires not only consideration of the antimicrobial properties of the ions but also the optimization function of the ions on the physicochemical properties of the material.

**Figure 3:**
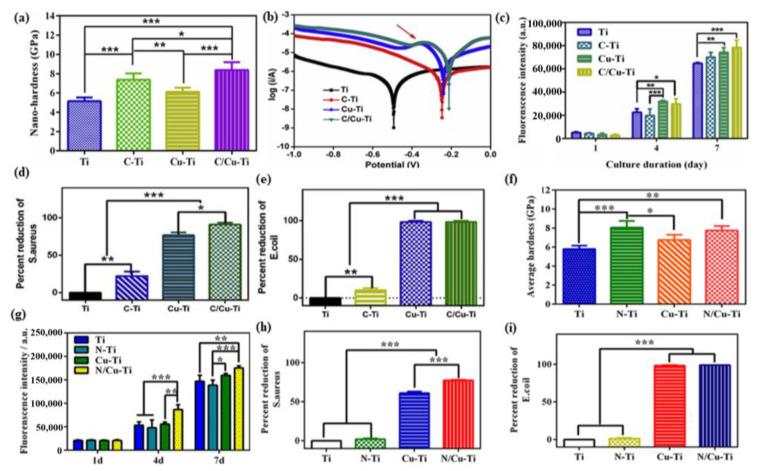
(**a**) Hardness average values (40–60 nm) detected by nanoindentation tests for various samples. (**b**) Polarization curves of various samples. (**c**) Proliferative activity of HUVECs cultured on various surfaces. Antibacterial rates of various samples against *S. aureus* (**d**) and *E. coli* (**e**). Reproduced with permission from Ref. [51]. Copyright 2020, KeAi Publishing LTD. (**f**) Hardness average values (40–60 nm) detected by nanoindentation tests for various samples. (**g**) Proliferative activity of HUVECs cultured on various surfaces. Antibacterial rates of various samples against *S. aureus* (**h**) and *E. coli* (**i**). *: *p* < 0.05, **: *p* < 0.01, ***: *p* < 0.001. Reproduced with permission from Ref. [53]. Copyright 2018, American Chemical Society.

Dual ion implantation can effectively improve the mechanical properties and corrosion resistance of Ti and reduce the cellular toxicity following the implantation of Cu. C/Cu co-implantation can effectively improve the mechanical properties of the Ti surface, and the formed Cu/C galvanic corrosion pair can improve the corrosion resistance of the Ti surface [51]. Figure 3a shows the nano-hardness curves. The C/Cu-Ti exhibits the largest nano-hardness value, followed by C-Ti, Cu-Ti and then Ti. Therefore, C/Cu ion co-implantation can significantly improve the nano-hardness values of Ti surfaces [51].

In contrast, the Cu implantation can only slightly increase the nano-hardness value of the Ti surface, probably due to the presence of internal metallic Cu [52]. Figure 3b shows polarization curves of the samples in physiological saline before and after modification, which suggests that the corrosion resistance of the modified samples was improved [51]. Similarly, N/Cu co-implantation can improve the average hardness (Figure 3f) and enhance the corrosion resistance of Ti materials [53].

The release of Cu ions was controlled by the galvanic effect, which effectively improved the resistance of the Ti surface to bacteria and showed good antibacterial effects against both *E. coli* and Staphylococcus aureus (*S. aureus*) [51,53] (Figure 3d,e,h,i). In addition, N/Cu-Ti [53] and C/Cu-Ti [51] have excellent biocompatibility with MC3T3-1 (Figure 3c,g). Moreover, Cu ion can improve the angiogenic properties [54] of the Ti surface and promote the proliferation and migration of HUVESCs. Other strategies, including Cu/Zn [46,55] ion co-implantation, can also improve the mechanical properties and antimicrobial capacity of the sample surfaces.

Overall, the co-implantation of multiple ions could become a promising direction to enhance the overall performance of Ti implants. For example, the co-implantation of copper ions with non-metallic ions can solve problems such as the reduced corrosion resistance of the material caused by the injection of copper ions. While the co-implantation of copper ions with other metal ions (e.g., Sr, Ca and Mg) could also enhance the functionality, such as the antibacterial activity as well as osteogenic and angiogenic properties. Conventional ion implantation technology often suffers from complicated operations and expensive costs. Research on ion implanted copper is mainly focused on plasma immersion implantation, while research on other ion implants, such as ion assisted deposition, is very rare and needs to be further developed.

### 3.2. Alloy

The main methods of alloy preparation include ingot metallurgy, powder metallurgy, selective laser melting etc. [56] Among them, arc melting [57,58,59] and powder metallurgy are the common methods for preparing alloys. The arc melting method involves introducing a certain percentage of the metal material into a clamp and then, after repeated evacuations, filling the vacuum furnace with protective argon gas. Afterwards, the plasma arc of the electrodes is heated to melt the elements completely, and then the entire melt is solidified into an alloy using rapid cooling with water.

The arc melting process is simple and has a wide range of applications, but the castings have disadvantages, such as component segregation, coarse structure and internal shrinkage. The powder metallurgy method uses metal powder as raw material and prepares the alloys by ball milling, mixing, extruding and sintering, which results in a more uniform alloy composition; however, the toughness of the casting is relatively poor. Zhang et al. [60] showed that the as-cast Ti-Cu alloy prepared by arc melting had high hardness and mechanical strength but lower corrosion resistance compared with pure Ti.

However, the hardness, strength and corrosion resistance of the samples were improved after thermal treatment (900 °C for 2 h), which had little effect on the antibacterial properties. The treatment of 900 °C/2 h + 400 °C/12 h further improved the hardness, corrosion resistance and mechanical strength and significantly improved the antibacterial effect. The Ti-Cu alloy prepared by the sintering process, on the other hand, had better corrosion resistance and hardness than the as-cast Ti-Cu alloy, as well as a better yield strength.

Compared to pure Ti, the Ti-Cu alloy had significantly higher hardness, yield strength and compressive strength [15,18], which can be explained by the solid solution strengthening of Ti and the fine precipitation of intra-metallic compounds that may be similar to dental silver amalgam [61,62]. Unlike the Cu-doped Ti surface prepared by ion implantation [50], the Ti-Cu alloy had improved corrosion resistance [15]. Table 1 summarizes the articles published on Ti-Cu alloys in recent years [15,35,37,39,40,60,63,64,65,66,67,68,69,70,71,72].

Ma et al. [63] showed that the Ti-Cu alloy with 5 wt.% Cu (Ti-5Cu) possessed better mechanical properties and corrosion resistance, as well as higher antibacterial activity against *E. coli* and *S. aureus* compared with pure Ti. Furthermore, the Ti-5Cu alloy had good cytocompatibility with no cytotoxic effects on MC3T3-E1 cells and superior hemocompatibility with low hemolysis rates. Liu et al. [39] found that the Ti-5Cu alloy exhibited antibacterial activity against Streptococcus mutans (*S. mutans*) and Porphyromonas gingivalis (*P. gingivalis*) through suppressing bacterial adhesion and biofilm formation.

**Figure 4:**
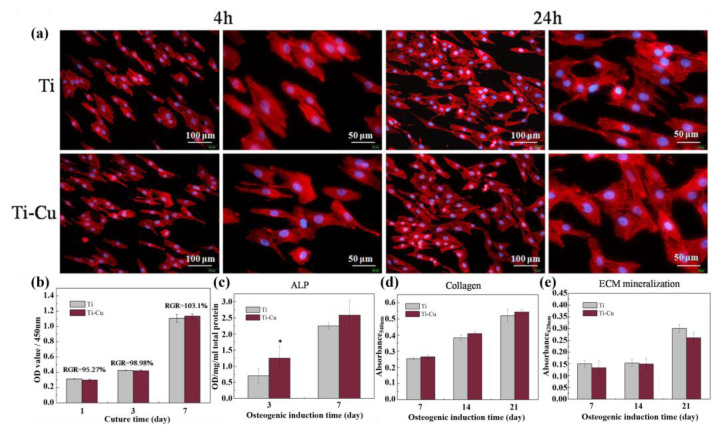
(**a**) Morphology of MG63 cells on the surfaces of Ti and Ti-Cu alloys. (**b**) Viabilities of MG63 cells determined by measurement of the optical density. Osteogenesis-related factors analysis. (**c**) ALP activity of cells on Ti and Ti-Cu alloy. * Ti-Cu alloy facilitated the early differentiation of MG63 cells. Quantitative colorimetric results of (**d**) ECM mineralization nodules and (**e**) collagen secretion. Reproduced with permission from Ref. [67]. Copyright 2019, Springer.

The Ti-5Cu alloy had no cytotoxicity to rat bone marrow mesenchymal stem cells (rBMSCs), and the released Cu^2+^ was substantially lower than the recommended daily intake of Cu. In 2019, Liu et al. [67] demonstrated the cytocompatibility and osteogenic ability of a Ti-5Cu alloy. The results of CCK-8 experiments revealed that the Ti-Cu alloy was not cytotoxic to MG63 cells (Figure 4b). In addition, the morphology of cells cultured on the surface of the Ti-Cu alloy was similar to that of cells cultured on the surface of Ti (Figure 4a), indicating excellent cell attachment and spreading on the material surface. Therefore, the Ti-Cu alloy had no negative effects on osteoblast adhesion, proliferation and apoptosis.

The Ti-Cu alloy promoted ALP activity (Figure 4c), indicating that the Ti-Cu alloy facilitated the early differentiation of MG63 cells. For ECM mineralization (Figure 4d) and collagen synthesis (Figure 4e), the Ti-Cu alloy had no significant effects in early and extended culture periods. However, for osteogenic-related genes, the Ti-Cu alloy significantly promoted osteogenic differentiation of MG63 cells by upregulating the expression of osteogenic-related genes, such as alkaline phosphatase (ALP), collagen I (Colla I), osteopontin (OPN) and osteocalcin (OCN).

Pure Ti and Ti-Cu alloy implants were artificially implanted in the mandibular premolar site of beagle dogs for 3 months and treated with ligature-infected treatment. The bone resorption of Ti implants was as high as 6.91 ± 0.60 mm, while that of Ti-Cu alloy implants was only 1.96 ± 0.84 mm, indicating that Ti-Cu alloy implants were effective in reducing bone resorption compared to Ti implants [35]. The anti-infective mechanism of Ti-Cu alloy with oral microbiological environment was also recently reported.

**Figure 5:**
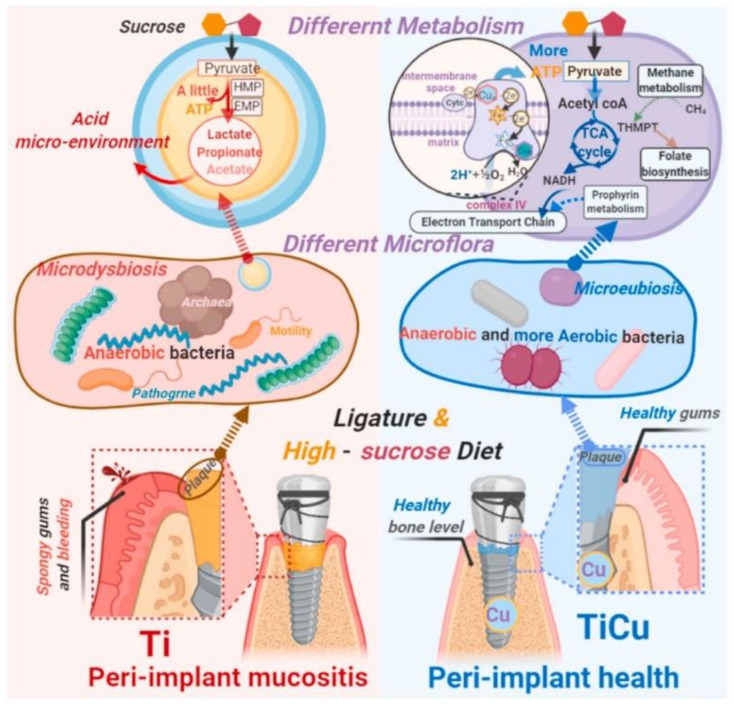
Schematic representation for anti-infective mechanism of TiCu implants in the ligature and sucrose-rich diet-induced model. Reproduced with permission from Ref. [38]. Copyright 2021, KeAi Publishing LTD.

Liu et al. [38] created a natural oral bacterial infection environment by a ligature dog model and sucrose-rich diet induction (Figure 5), and sucrose-rich diets affected endogenous species of the Ti and Ti-Cu implant surface microbiota, resulting in significant differences in carbohydrate metabolism. In the plaques of Ti implants, anaerobic bacteria produce acids that acidify the microenvironment, accelerating the accumulation of acid-producing bacteria and pathogens.

As the ecological disorders of the oral microenvironment occur, bacteria colonize and infect the tissues around the implants, eventually causing disease around the implants in animals [38]. In contrast, Ti-Cu implants promoted oral flora carbohydrate metabolism through the TCA cycle, maintaining metabolic balance and avoiding acidic plaque formation. Ti-Cu implants also maintained the balance among anaerobic and aerobic bacteria, salivary microbiota and the health of the peri-implant area [38]. Therefore, alloying Ti implants with Cu is a promising strategy to enhance the antimicrobial properties of Ti implants.

Zhang et al. [15] prepared a new antibacterial Ti-Cu alloy by adding Cu to pure Ti using a powder metallurgy technique. This Ti-Cu sintered alloy showed excellent antibacterial effects against *S. aureus* and *E. coli*, as well as significantly higher hardness and slightly improved corrosion resistance compared with pure Ti. However, the Ti-Cu sintered alloy only inhibited the bacteria in contact with the alloy, suggesting that its antibacterial properties may be related to the release of Cu ions.

According to the results of acridine orange/ethidium bromide (AO/EB) fluorescence analysis, the cell death of both pure Ti samples and Ti-Cu samples could be attributed to apoptosis rather than necrosis, without evidence of correlation between apoptosis and Cu content [37]. MG63 cells showed good cell adhesion and spreading on Ti-Cu alloys with different Cu contents, and the increase of Cu content had no effect on cell survival [37].

There was no significant difference in the differentiation of MG63 cells between pure Ti samples and Ti-Cu sintered alloys with different Cu contents [37]. Wang et al. [36] investigated the in vivo antibacterial properties of Ti-10Cu sintered alloys against *S. aureus*. The pure Ti implants showed severe postoperative infection, inflammation and suppuration, while the Ti-10Cu implants showed only minor infection after 4 days of surgery, demonstrating the strong in vivo antibacterial properties of the Ti-10Cu sintered alloy.

Additional surface modification of Ti-Cu alloys can further enhance the performance. Liu et al. [68] prepared SLA-TiCu surfaces on Ti-Cu alloy surfaces using sandblasting and large-grits etching (SLA) techniques and found that the SLA-TiCu surfaces significantly promoted osteogenic differentiation compared to other surfaces (Ti, Ti-Cu, SLA-Ti). In addition, the SLA-TiCu surface has a better antibacterial effect, which may be attributed to its serrated surface structure on the one hand and its hydrophobicity on the other.

Compared with other surfaces, the SLA-TiCu surface showed a slight decrease in cell adhesion at the early stage, which may also be caused by its hydrophobicity. Liu et al. [69] demonstrated that SLA-TiCu surface has a better osseointegration and anti-infection ability in vivo using a beagle model, demonstrating the positive effect of SLA-TiCu on the angiogenesis of HUVECs. In addition, the surface bioactivity could also be improved by anodic oxidation without affecting the antibacterial properties [73].

This research illustrates the potential clinical application of combining multiple surface modification methods to enhance surface bioactivity. Indeed, the combination of Ti-Cu alloy with other alloys or application of multiple surface modification strategies to the Ti-Cu alloy would become a popular direction, yet currently the relevant research is considerably less. In addition, except for the arc melting and powder metallurgy methods, there is less research on other alloy preparation methods, such as 3D printing and spark plasma sintering, which are promising technologies and should be further explored.

### 3.3. Electrochemical Techniques

Common electrochemical treatment techniques include electrodeposition, anodic oxidation, micro-arc oxidation etc. The electrodeposition method is used for electroplating. Typically, two metal electrodes are immersed in a specific electrolyte and an external electric field is applied to deposit the required metal on the working electrode. Electrodeposition has proven to be one of the most versatile methods for preparing nanostructured coatings [74], with features that are superior to conventional deposition techniques (e.g., low processing temperatures, low-cost equipment, possibility of fabrication on porous substrates with complex shapes and simple control of coating properties) [75,76].

**Figure 6:**
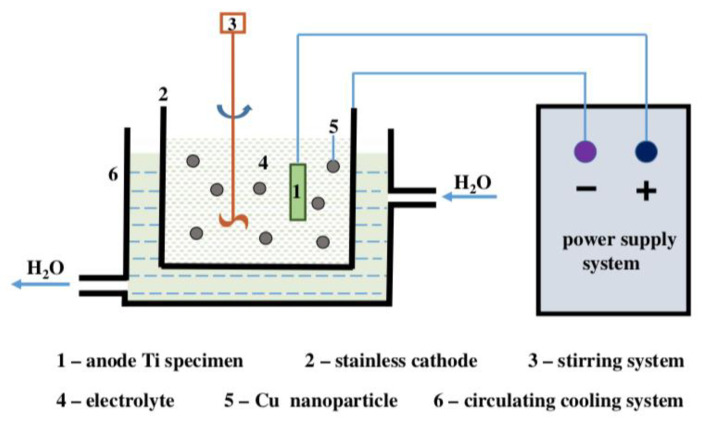
Schematic diagram of the MAO experimental device. Reproduced with permission from Ref. [34]. Copyright 2018, Elsevier.

Micro-arc oxidation (MAO), also known as plasma electrolytic oxidation (PEO), was derived from anodic oxidation technology. It uses arc discharge to enhance and activate the reaction occurring on the anode to form a high quality reinforced ceramic film on the surface of metal substrate (Figure 6). The micro-arc oxidation film has the characteristics of strong adhesion with matrix, compact structure, high toughness, good wear resistance and corrosion resistance.

MAO produces a hard and thick porous TiO_2_ coating and, as the voltage rises during the generation of the TiO_2_ film, a large number of micro-arc discharges break through the oxide film. The target ions and oxygen within the electrolyte then enter the inner regions of the coating through these discharge channels, resulting in a coating doped with bio-functional ions (e.g., Zn, Ca, Cu, P, Ag and Bi) [77,78,79,80].

Many studies have demonstrated the superiority of MAO technology for treating Ti-based materials for medical devices. MAO coatings doped with calcium (Ca) and phosphorus (P) can improve the biocompatibility of Ti substrates [81,82,83,84]. Due to the good properties of Cu ions, such as the antibacterial properties exhibited at low concentrations with low cytotoxicity [85], many researchers have used the MAO technique to incorporate Cu ions together with other bio-functional ions onto Ti surfaces in one step to prepare antibacterial coatings, and the effectiveness of this approach has already been proven.

Table 2 summarizes reported papers on the one-step incorporation of Cu to Ti surfaces using the MAO technique [30,32,33,34,86,87,88,89,90,91,92,93,94,95]. Notably, surfaces treated with MAO of Ti substrates using electrolytes containing Cu have been shown to have antibacterial effects against a wide range of bacteria, including *E. coli*, Methicillin-resistant Staphylococcus aureus (*MRSA*), *S. aureus* and *S. mutans*. The Cu content in TiO_2_ coatings would affect the antibacterial properties and cytotoxicity.

Zhang et al. [33] reported that microporous TiO_2_ coatings doped with appropriate doses of Cu exhibited remarkable antibacterial properties. The coating surface containing different copper contents is uniformly distributed with micro-pores of 1–4 μm in diameter. The doping of copper elements does not change the structural characteristics of the coating, including the morphology, size and distribution of the pores. With the gradual increase of Cu^2+^, the surface wettability decreased significantly, which may be directly related to the change in the content of hydrophilic groups (such as basic Ti-OH) on the surface.

Compared with pure TiO_2_ coatings, the adhesion and proliferation of fibroblasts could be significantly enhanced by the TiO_2_ coating loaded with 0.63 ωt% Cu and obviously inhibited with 1.93 ωt% Cu. Implant surfaces could play an important role in preventing infection and promoting osseointegration, depending on their antimicrobial properties and whether they are harmful to osteoblasts.

Thus, there is a need to recognize the importance of balancing the antimicrobial properties of Ti surfaces with biocompatibility. Zhang et al. [34] showed that Cu nanoparticles coatings can exhibit good antibacterial activity, with low Cu levels (0.3 Cu) promoting osteoblast proliferation and adhesion, while high Cu levels (3.0 Cu) are significantly cytotoxic, consistent with what was previously reported by Hang et al. [96].

**Figure 7:**
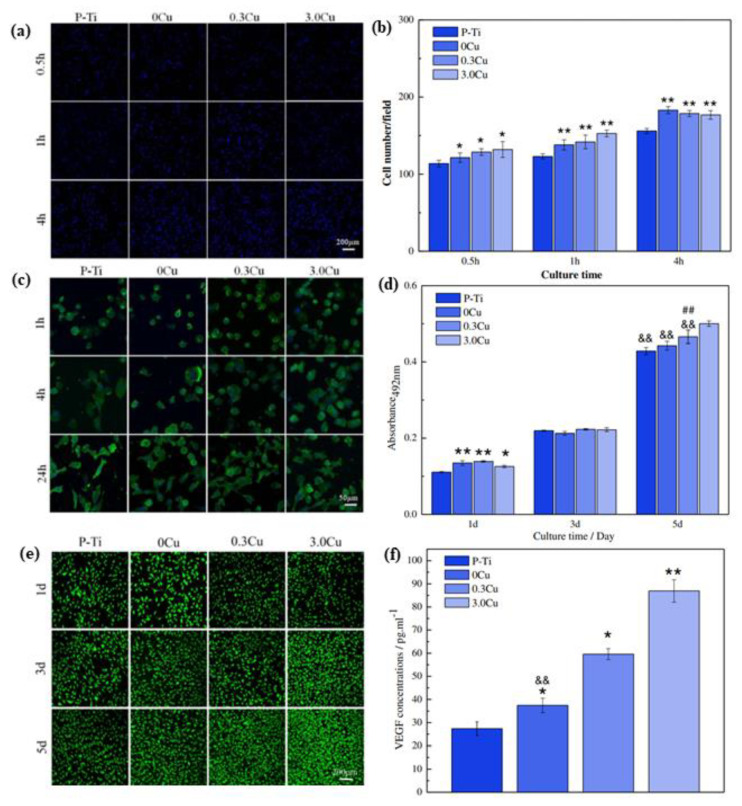
Response of endothelial cells: (**a**) fluorescence images and (**b**) quantitative results of cell adhesion; (**c**) fluorescence images of endothelial cells cultured on the MAO coatings with F-actins stained with FITC (green) and nuclei stained with DAPI (blue); (**d**) fluorescence images of live/dead staining of endothelial cells on the MAO coatings; (**e**) MTT results of endothelial cells on the MAO coatings; and (**f**) VEGF concentrations secreted by endothelial cells. **: *p* < 0.01 compared to P-Ti, ##: *p* < 0.01 compared to 0 Cu, &&: *p* < 0.01 compared to 3.0 Cu, *: *p* < 0.05 compared to P-Ti. Reproduced with permission from Ref. [34]. Copyright 2018, Elsevier.

Therefore, appropriate doses of Cu can promote the up-regulation of osteogenic-related proteins, such as ALP, OCP and OCN in osteoblasts and bone mesenchymal stem cells [33]. However, in the case of endothelial cells, 3.0 Cu could facilitate cell proliferation and adhesion to promote angiogenesis [34,97]. The initial adhesion of endothelial cells was detected as shown in Figure 7a,b. The number of adherent endothelial cells on MAO coating was higher than that on Pure-Ti (P-Ti) coating throughout the incubation, and the number of adherent cells did not change significantly as the incubation time increased from 0.5 to 4 h.

The cell spreading activity is shown in Figure 7c. The cell morphology on the surface of 0.3 Cu and 3.0 Cu was more dispersed, with a higher number of filopodia and lamellipodia. It can be inferred that Cu NPs doped with TiO_2_ coating is more favorable to the spreading of endothelial cells, and the higher the Cu concentration, the better cell spreading. MTT result (Figure 7d) shows that the cell numbers increased with time, and the order of cell numbers after 5 days was: 3.0 Cu > 0.3 Cu > 0 Cu > P-Ti, indicating that Cu NPs can promote endothelial cell proliferation and are not cytotoxic. The live/dead stained fluorescence images of endothelial cells are shown in Figure 7e.

The results are in agreement with the MTT results. Endothelial cells were most viable on the surface of 3.0 Cu. ELISA analysis (Figure 7f) shows that the MAO coating could promote vascular endothelial growth factor (VEGF) secretion compared to P-Ti. More than that, the addition of Cu NPs to the TiO_2_ coating could further promote the secretion of VEGF with the increase of Cu concentration. Huang et al. [32] demonstrated that Cu-doped MAO coatings enhanced macrophage-mediated osteogenesis and bactericidal capacity and, to some extent, promoted inflammatory responses.

These research results provide new ideas for the design and application of Cu-doped Ti biomaterials. In addition to enhancing the surface osteogenic and antimicrobial activity, Cu is expected to be used as an additive to promote angiogenesis and as a modulator to balance macrophage-mediated bone regeneration and bactericidal capacity. Although Cu can promote osteogenesis and angiogenesis, fewer species of bacteria can be killed by Cu than by Ag.

Gallardo-Godoy et al. [98] deposited Ag coatings in oxidized and metallic states on titanium surfaces using anodic oxidation techniques, which significantly reduced in vitro bacterial adhesion of Streptococcus sanguinis (*S. sanguinis*) and Lactobacillus salivarius (*L. salivarius*), while maintaining a good in vitro biocompatibility. Further studies on the effect of copper on other bacteria are still needed. In addition to controlling the loadings, the Ti surface can also be effectively modified by adjusting the MAO parameters.

Prosolov et al. [99] deposited CaP-based coatings containing Zn and Cu obtained by the MAO process on pure titanium and Ti-40Nb alloy substrates by adjusting the voltage in order to control the surface crystallinity and crystallinity. In addition, the application of MAO technology to co-dope Cu with other elements (e.g., Zn/Cu [88], Mg/F/Cu [90], Si/Cu [91]) into the Ti surface in one step can also enhance the overall performance of the coatings, while the synergistic effect of these ions on bacteria and cells in vitro and in vivo remains to be further explored.

Cu-containing surfaces can be also prepared by plasma electrolytic oxidation combined with electrodeposition on Ti6Al4V plates [17]. First, a porous Ti oxide layer is formed by plasma electrolytic oxidation, and then Cu ions were galvanically deposited onto the surface. The obtained Cu/Ti oxide composite layer was sandblasted using glass spheres to obtain a surface with an average Cu loading of 1 mg/mm^2^ [17].

As far as the coating is concerned, the Cu ions released from the surface can effectively kill planktonic and adherent *S. aureus*. The appropriate concentration of Cu^2+^ (0.05–0.3 mM) can promote not only the proliferation of MSCs but also the osteogenic differentiation of MSCs [17]. However, when the Cu^2+^ concentration was 0.5 mM, there was a significant toxic effect on MSCs. This may explain the contradictory conclusions drawn by using osteoblast cell lines, and these results show both inhibitory and stimulatory effects of Cu on the proliferation of MSCs [100,101].

Hence, the functional activity of the Cu ions that released from the surface depends on the time and the distance of the tissue to the implant surface [17]. After implantation, high Cu ion concentrations in the immediate vicinity of the implant surface can produce antimicrobial effects. In contrast, at greater distances from the implant surface or when the Cu reservoir on the material surface is gradually exhausted, MSC proliferation and differentiation can be stimulated due to lower Cu ion concentrations.

Prinz et al. [102] used Cu-containing coated nails prepared by galvanic deposition for the fixation of tibial fractures in a rabbit model and then inoculated with bacteria. After implantation, the Cu ion concentration did not increase in the blood, which indicates that the release of Cu ions from the implant was exclusively confined to the fracture site. Moreover, the Cu ions released by the implants can prevent not only the reversible attachment of bacteria but also the irreversible attachment of bacteria, i.e., the formation of a biofilm, which indicates that coating implants with Cu is a suitable strategy for the prevention of associated infections.

In addition, the X-ray examination showed an increase in the bone crusting index in animals implanted with Cu-coated nails, suggesting that the Cu ions released by the coating could promote bone formation [102,103]. Due to this functional property, Cu-doped Ti implants prepared by galvanic deposition have great promise for applications. However, the film layers prepared by electrodeposition are inevitably subjected to stress problems. The high internal stress often causes abrasion cracking. Therefore, research efforts should be devoted to eliminating the coating stress and to improving the stability.

### 3.4. Sputtering

Magnetron sputtering is a process where atoms or molecules are ejected from a target by bombardment of high-energy plasma, which then travel through the vacuum environment and deposit onto a substrate. During the sputtering process, the electrons on the target surface are accelerated under the action of an electric field and collide with the sputtering gas Ar to produce argon ions and secondary electrons [104]. The magnetron sputtering technique is capable of preparing thin films with strong bonding to the substrate, and the preparation conditions are simple and controllable, which can avoid the defects and adverse effects caused by chemical methods during the preparation process [105].

The formation and growth of thin films are strongly influenced by the plasma parameters and energy (particle) flux [106,107]. Conventional direct current magnetron sputtering (dc-MS) is typically characterized by low ionization and ion flux [108,109]. The main features of high-power pulsed magnetron sputtering (HiPIMS) operating at low frequencies (~100 Hz) and short pulse widths are higher ionization of sputtered particles and higher ion fluxes [110,111]. Dual sputter source high power pulsed magnetron sputtering (dual-HiPIMS) can be used for the deposition of multicomponent films and alloys [112].

Stranak et al. [113] applied the above three different techniques to deposit Cu-containing films with different chemical compositions on Ti6Al4V substrates. Generally, these films are composed of Ti and Cu metals; however, a few oxides are also formed on the surface of the films. The films with different releases of Cu ions could be achieved by Dulbecco’s Modified Eagle’s Medium (DMEM), where the dual-HiPIMS technique produces films with higher Cu content and density and can be completely released in DMEM [113]. Among these films, only the films prepared by dual-HiPIMS technique showed antibacterial effects against the planktonic bacteria S. epidermidis and *S. aureus*, which may be related to the rapid release of Cu ions.

The antibacterial effect of Cu-doped films prepared by the dual-HiPIMS technique was better than that of Cu-doped surfaces prepared by the plasma immersion injection technique [114], which may also be related to the rapid release of Cu ions. This short-term release of Cu ions also affects the growth of surface osteoblasts. MG63 cells were grown on the films generated by the dual-HiPIMS technique, and a significant increase in osteoblast viability was observed when Cu ions were completely released [113].

To explore the factors affecting the release of Cu ions from thin films, Stranak et al. [115] deposited two layers of films at different pressures using the high-power impulse magnetron technique. The ion energy is the main factor affecting the formation of crystalline films, while the ion energy is strongly influenced by the pressure. At higher pressures, i.e., lower ion energies into the matrix, grain-like structures with larger crystals (domain size of about 10–20 nm) would be formed.

Conversely, high-energy ion bombardment, observed at low pressure, formed smooth, dense films and small Cu crystals of about 2 nm. The release of Cu ions from Ti-Cu films into DMEM depends mainly on the structure of the deposited film. The grain-like structure increases the effective contact area with the DMEM incubation medium, which is responsible for the rapid and intensive release of Cu ions. On the other hand, only smooth and dense films prepared at low pressure react with DMEM, leading to moderate Cu release [115].

**Figure 8:**
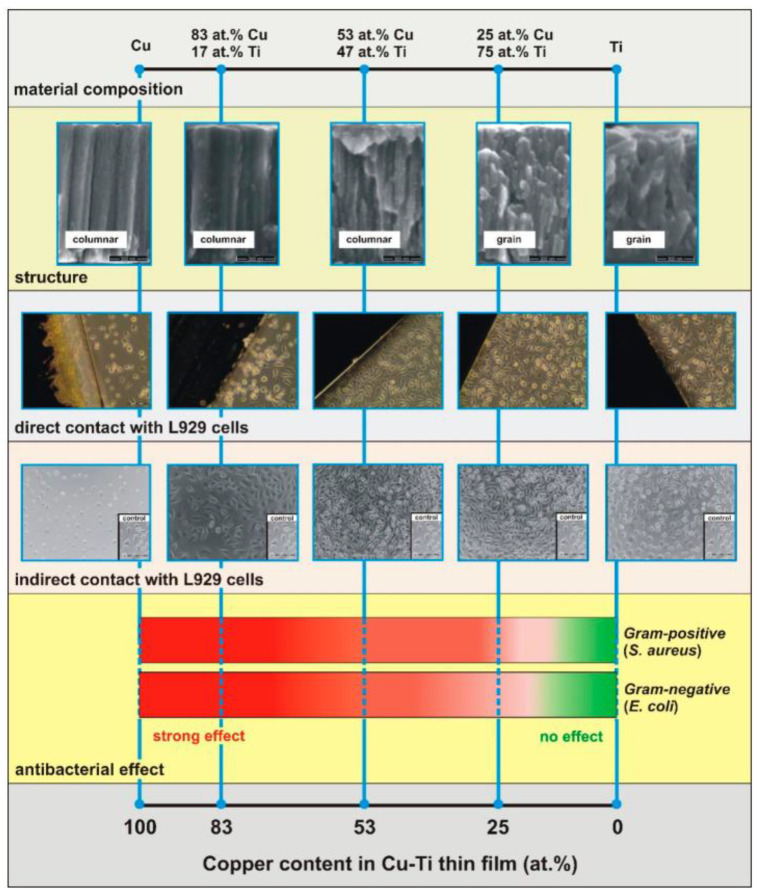
The influence of material composition on the biological properties of nanocrystalline thin films based on Cu and Ti. Reproduced with permission from Ref. [114]. Copyright 2020, Multidisciplinary Digital Publishing Institute.

Wojcieszak et al. [116] prepared Cu-doped nanocrystalline coatings (Figure 8) using the pulsed DC magnetron sputtering technique. There was a significant change in the structure of Cu-Ti films with the increase of Cu content. The sample with 25 at% Cu doping (Cu25Ti75) showed a strong antibacterial effect on both *E. coli* and *S. aureus* compared with pure Ti film. The Cu content in Cu-Ti films had a significant effect on the physiological function of mouse fibroblasts (L929).

Direct or indirect contact with Cu83Ti17 or Cu films resulted in fibroblast cell death; however, no significant cytotoxicity was observed when the Cu content was less than 53 at%. At the same time, Cu25Ti75 films had no effect on inducing fibroblast cell death in mice, did not interfere with the cell cycle and showed cell promoting properties in wound healing experiments [117]. It is worth mentioning that the Cu ion release from Cu25Ti75 films was only 0.003 ppb/mm^2^, a dose that is not sufficient to produce indirect cytotoxic effects on eukaryotic cells (for mouse fibroblasts L929) but sufficient to obtain good antibacterial effects (for *E. coli* and *S. aureus*).

Milan et al. [118] prepared Cu/a-C:H composite multifunctional coatings on Ti6Al4V alloy surface by magnetron sputtering technique in a mixture of Ar and CH_4_ gas. By adjusting the ratio of Ar/CH_4_ in the gas mixture, the surface structure, corrosion resistance and mechanical properties of the films could be changed. When the Ar/CH_4_ ratio was 2.3, the film thickness, adhesion to the substrate and impermeability were optimal, and the corrosion resistance was the highest, meeting the hardness test requirements.

The Cu/a-C:H coated Ti implant had a better antibacterial effect against *P. gingivalis*, as well as enhanced angiogenesis and osteogenic activity. This study demonstrated the importance of a controlled atmosphere and film microstructure for the biocompatibility of the modified surfaces when magnetron sputtering is used for surface modification.

In addition, the surface microhardness and wear resistance of the Ti-Cu-N coatings obtained by combining magnetron sputtering with plasma nitriding to modify the material surface were significantly improved, along with the good corrosion resistance of the coatings [119]. He et al. [120] prepared TiO_2_/CuO coatings on Ti surfaces by combining magnetron sputtering and annealing. Compared with Ti and TiO_2_ coatings, the TiO_2_/CuO coating had superior corrosion resistance and antibacterial ability against Staphylococcus aureus without significant cytotoxicity, and this also promoted the diffusion and proliferation of MC3T3-E1 cells.

Radio frequency magnetron sputtering is also a common sputtering method that offers better control of the coating morphology and structure, as well as a high adhesion of the coating to the substrate [121]. The coatings for implants should not delaminate or form any debris during implantation. Prosolov et al. [122] prepared Cu-substituted hydroxyapatite coatings using the RF sputtering method with tilting angles. At the oblique angle, the film presents a distinct directional nanostructure, while the oblique deposition can control the structure and nano-roughness of the coatings.

They prepared novel bioactive and antibacterial RF coatings with enhanced osseointegration properties by depositing Zn-HA or Cu-HA coatings on Ti-6Al-4V and Ti-6Al-7Nb alloys [123]. The deposited coatings are amorphous and have a calcium-rich elemental composition. RF magnetron sputtering was combined with the annealing process to crystallize amorphous CaP coatings by thermal annealing under an ambient atmosphere. The coating extracts exhibited in vitro inhibition against the pathogenic strain 209P, the causative agent of *S. aureus*.

Not only that, the RF CaP films with the addition of Zn or Cu ions also have good osteointegration properties. Application of the magnetron sputtering technique to introduce Cu on a Ti surface can effectively improve the corrosion resistance and mechanical properties of the implant surface, and on this basis—combined with plasma nitriding, plasma immersion implantation, annealing treatment and other techniques—the coating can exhibit excellent biocompatibility, which may have potential application in the field of biomedical implant materials.

### 3.5. Sol-Gel

The Sol-gel method uses compounds that contain highly chemically active components as precursors, which are uniformly mixed under the liquid phase and then undergo hydrolysis and condensation chemical reactions to form a stable and transparent sol system in solution. The solute is slowly polymerized between the aged gel particles to create a three-dimensional network structure of the gel, and the gel network is filled with a solvent that loses its fluidity to form the gel. The gels are dried, sintered and cured to prepare molecular or nanostructured materials.

Cu-doped TiO_2_ monolayer and multilayer coatings on Ti6Al4V alloy substrates and CuO/TiO_2_ composite nanofibers were obtained using the sol-gel method [124,125]. In the former, titanium isopropoxide was used as the Ti precursor, copper (II) acetate hydrate and Cu powder as the Cu precursor [124]. The latter results in nanofibers with good morphology, while the former has a negative effect on the morphology of the nanofibers, and the one-dimensional structure of the coating was completely destroyed after calcination [124].

The CuO/TiO_2_ nanofibers prepared with Cu powder have anatase and rutile phases, while the CuO present in the Cu-containing nanofibers has excellent crystallinity. CuO/TiO_2_ nanofibers showed good antibacterial activity against Klebsiella pneumoniae, and the inhibitory effect on Klebsiella pneumoniae was enhanced with the increase of nanofiber concentration [124]. Higher concentrations of CuO/TiO_2_ nanofibers showed excellent toxicity against the tested pathogenic strain [124]. The latter used copper nitrate and titanium isopropoxide as the precursors of CuO and TiO_2_, respectively, to prepare CuO/TiO_2_ nanorods using the electrostatic spinning process [125].

With a diameter of 100 nm and an average length of 1 μm, composite CuO/TiO_2_ nanorods exhibit stronger antibacterial activity against *E. coli* and *S. aureus* under visible light compared with TiO_2_ [125]. In addition, the nanoscale of CuO/TiO_2_ nanorods can improve their antibacterial properties due to their large surface area. Moreover, Cu-doped TiO_2_ was prepared using sol-gel chemistry with different concentrations of copper acetate solutions (3, 6 and 9 ωt%) as precursors, and the addition of Cu enhanced the crystallinity and reduced the grain size of the products [126].

The inhibition zones were recorded as (0.8–3.25 mm) and (2.55–4.85 mm) for *S. aureus* for the doped low and high doses of Cu, respectively, while both low and high doses were ineffective for *E. coli* [126]. It can be seen that Cu-doped TiO_2_ had a stronger bactericidal and synergistic effect on *S. aureus* compared to *E. coli*. In addition, the multi-walled carbon nanotubes on the surface of Ti-based implants coated with fluorapatite-titanium alloy were decorated using a sol-gel method using copper acetate as a Cu precursor. The obtained dental implant coatings had good morphological characteristics, and the doping of Cu improved the nanomechanical properties of the composite coatings [127].

Moreover, a Cu/TiO_2_ (CTD) nanocomposite with an average particle size of 10 nm was obtained by reducing the precursor of copper chloride in TiO_2_ colloids using a 400 W high-pressure Hg lamp light source [128]. The nanocomposite had an excellent crystallinity and MICs of 21.4 and 14.3 μg/mL for *E. coli* and *S. aureus*, respectively. In addition, the MBCs of CTD against *E. coli* and *S. aureus* were 23.9 and 17.1 μg/mL, respectively, which were much lower than those previously reported for Ag [129,130], Cu [129], Cu_2_O [131] and CuO [131] nanoparticles.

However, the sol-gel method involves a large number of process variables in the reaction process, which may compromise the functionality of the material. In addition, the raw material cost of the sol-gel method is high, and some of the raw materials are organic, which is harmful to health and not conducive to industrial applications. Therefore, how to reduce the cost and simplify the synthetic procedure is of great importance for the industrial application of the sol-gel method in implants.

## 4. Conclusions and Perspectives

The modification of Ti and Ti-based implants for the purpose of preventing prosthetic infections is challenging research. The application of Cu as an antimicrobial agent directly grafted onto the implant surface can prevent bacterial colonization. Together with topical medication, it can effectively prevent or treat infections and overcome the growing problems of bacterial resistance to antibiotics. Cu-doped Ti-based implants have great potential for future applications due to their strong antimicrobial and bone-enabling abilities as well as their good corrosion resistance and mechanical properties. However, there are certain challenges regarding the clinical applications of this strategy.

1. Combination of different surface modification methods. The above-summarized surface modification methods have pros and cons.
  (1) The process of ion implantation is more complex and thus difficult to operate but results in little damage to the material surface and does not change the original size and roughness of the implants. Therefore, it is very suitable for the processing of precision substrates. Due to its high strength and corrosion resistance, titanium alloy has been widely used in clinical applications. However, its wear resistance is poor, and the corrosion products of particles entering human tissue after wear may lead to implant failure. The ion implantation technology can effectively strengthen its surface wear resistance.
  (2) The MAO technology has the advantages of simple and fast processing process; however, its high energy consumption leads to high commercialization costs.
  (3) The magnetron sputtering coatings have a superior bond with the substrate, the coating thickness can be tuned by adjusting the process parameters, and the co-sputtering of different metals can be realized, which is suitable for industrialization. However, it faces problems, including low target utilization and difficulty in sputtering magnetic targets.
  (4) Although sol-gel methods easily achieve doping at the molecular level, they are expensive in principle and time consuming, which will increase the cost of commercialization. Overall, there is a trend to use different surface modification methods simultaneously to achieve better antibacterial effects and to promote osseointegration—for example, Ti-Cu alloys with combined sandblasting and acid etching technology and Ti-Cu alloys with combined anodic oxidation technology, magnetron sputtering and ion implantation technology.
  In addition to the above-mentioned modification strategies, there are also hydrothermal methods, ion exchange methods and chemical vapor deposition that can effectively dope copper onto the titanium surface. Based on this, the development of a commercial titanium-based surface with excellent antibacterial effects that enhances tissue integration will be possible. Future research in the field of biomaterials should be directed toward combining multiple surface modification processes to provide long-term antimicrobial effects and to promote tissue integration.
2. Bactericidal ability and potential toxicity of Cu. Cu has been proven to be an effective antibacterial agent, and Cu-incorporated coatings show excellent antibacterial activity against *S. aureus* [15,36], *E. coli* [60,64], *S. mutans* [39] and *P. gingivalis* [68,69]. When the concentration of Cu^2+^ was 5 × 10^−5^ M, the bactericidal rate of Staphylococcus aureus was 92%, whereas when the concentration of Cu^2+^ was 5 × 10^−6^ M, the bactericidal rate of *E. coli* was 93% [132,133].
  Miyano et al. [134] evaluated the antibacterial activity of some pure metals using plate counting. The results of plate counting after 24 h incubation showed that the antibacterial effects from high to low were: Pb > Cu > Co > Zn > Ni > Zr > Mo. Compared with other metals, copper has better antibacterial effect and biocompatibility. Numerous in vitro tests have demonstrated the low ion release of Cu-doped Ti implants prepared by various methods and their excellent cytocompatibility without cytotoxicity to MG63 [15,37], MC3T3-E1 [56,71], L929 [33,88] and other cells.
  It was reported that the median toxic concentration of Cu ions on human gingival fibroblasts was 21.86 mg L^−1^ [135], and when the Cu ion concentration was higher than 9 mg/L, it was cytotoxic to MC3T3 cells [136]. Although the high concentration of Cu is thought to be toxic to mammalian cells, the concentration of Cu ions released from the surface of the Cu coating is low enough that the cytotoxicity is negligible.
  The effective concentration of antibacterial activity was much lower than that of cytotoxicity. However, the studied surface Cu ion release has certain problems, such as ion burst release (usually reaches a maximum within one day and then decreases rapidly in the next 10–30 days), and the antimicrobial effect increases with Cu ion concentration; however, there is potential cytotoxicity to cells when the Cu ion concentration is too high, which may cause long-term toxicity or side effects in humans.
3. The antibacterial mechanism of Cu ions has yet to be studied. It is widely believed that metal ions can kill bacteria by inducing the production of reactive oxygen species (ROS) [137]. ROS are the oxygen reduction products, such as peroxides, superoxides, hydroxyl radicals and singlet state oxygen. However, many studies have shown that Cu-doped Ti-based implants still have antimicrobial effects when the concentration of Cu ions released is very low. For example, the release of Cu ions from the antimicrobial Ti6Al4V-5Cu alloy was 2.498 ± 0.755 μg/L after 20 days of immersion in 0.9% NaCl solution [138].
  This is due to the ability of Cu-containing particles to resist bacterial adhesion [138,139] and biofilm formation [35], which would kill bacteria on the surface. However, the exact antimicrobial mechanism in the contact sterilization mode is not known. Moreover, electron transfer in bacterial activity is another widely accepted antibacterial mechanism [140,141,142]. Although many antimicrobial mechanisms have been studied in detail, the antimicrobial mechanism of Cu-doped Ti-based implants is still not fully understood.
4. Surface biological activity. The current research on Cu has focused on its antimicrobial effects, and there is a lack of research on whether the elemental Cu can enhance cellular and tissue activity. Although it has been shown that Ti-Cu alloys can promote the osteogenic differentiation of MG-63 cells by increasing the expression of osteogenic-related genes, such as ALP, Collagen I, OPN and OCN [67], few reports exist on the effects of cellular and tissue inertness of Cu-doped Ti implants prepared by other preparation methods on cells and tissues. It is also unclear whether a decrease in antimicrobial effects accompanies the increase in surface bioactivity. Thus, the effect of loading Cu on the implant surface in different ways on the cellular/tissue response and antimicrobial activity remains to be investigated.

Due to these problems, there are currently no Ti-based implants with Cu elements available in the medical device market. However, the problem of prosthetic infection is a crucial issue for dental and orthopedic implants. The combination of multiple antimicrobial elements and contributing bone elements, such as Ag^+^ [143,144], Cu^2+^ [15,33,39], Zn^2+^ [145] and Sr^2+^ [146,147], is a future trend. Future research in the biomaterials field should include the development of innovative metal surfaces that can improve and enhance tissue integration and provide long-lasting antimicrobial effects.

## Figures and Tables

**Table 1:** Preparation technology and related properties of Ti-Cu alloys.

Technique	Release	Tested Bacteria	Biocompatibility	Refs.
Powder Metallurgy	In 0.9% NaCl up to 72 h (0.05 mg/L)	*S. aureus* and *E. coli*	MG63	[15,37]
Arc Melting	In 0.9% NaCl up to 24 h (7–27.5 μg/L)	*S. aureus*	No report	[60,64]
Laser Powder Bed Fusion	No report	*S. aureus* and *E. coli*	No report	[65]
Selective laser melting	In a study solution (pH = 2.3) consisting of (10.0 ± 0.1) g/L 90% C_3_H_6_O_3_ and (5.85 ± 0.005) g/L NaCl for 7 days ± 1 h (0.90 μg/cm^2^)	No report	RAW264.7 and HUVEC	[66]
Selective laser melting	In the Hanks’s solution for 24 h (40–90 μg/L)	*E. coli*	MG63	[70]
Arc Melting	In 5 mL of PBS in a humidified atmosphere containing 5% CO_2_ for 7–28 days (2–15 ng/mL)	*S. aureus* and *E. coli*	MC3T3-E1	[71]
Spark plasma sintering	In artificial body fluid for 1–28 days (0.4–1.6 μg/mL)	*S. aureus* and *E. coli*	No report	[72]
Arc Melting	In 0.9% NaCl solution for 1, 4, 7, 14, 21, and 35 days (substantially below the recommended daily intake of Cu, 3–7.5 μg/L)	*S. mutans* and *P. gingivalis*	rBMSCs	[39]
Arc Melting	In NS for 1–30 days (the rate of 8.3 μg/L per day in the first 10 days and 2.36 μg/L per day in the subsequent 20 days)	*S. aureus* and *E. coli*	MC3T3-E1	[63]
Arc Melting	In 0.9% NaCl up to 24 h (3 μg/L)	*S. aureus* and *E. coli*	MG63	[35,67]
Arc Melting then treated by sandblasting and large-grits etching	In 0.9% NaCl solution for 1, 3, 7, 14, and 21 days (after 21 days reached 83.5 μg/L)	*S. mutans* and *P. gingivalis*	MC3T3-E1	[68,69]
Arc Melting	In PBS for 7, 14, 21, 28, 35, and 42 days (the rate of Cu^2+^ release was calculated as 0.106 mg/cm^2^/d)	*MRSA*	No report	[40]

**Table 2:** Summary of the recent papers related to the incorporation of Cu elements into Ti surfaces by the one-step MAO.

Year	Elements	Tested Bacteria	Cell Culture	Ref.
2016	Cu Cu	No report *S. aureus*	No report L-929	[86] [33]
2017	Cu	No report	No report	[87]
2018	Cu Cu and Zn Cu	*S. aureus* *S. aureus* *S. aureus*	RAW 264.7 and SaOS-2 L-929 MC3T3-E1 and Endothelial cell	[32] [88] [34]
2019	Cu Mg, Cu and F	No report *S. aureus*	No report MC3T3-E1	[89] [90]
2020	Cu and Si Cu or Ag Cu Cu, Zn, and P Cu	*S. aureus* and *S. mutans* *E. coli* *S. aureus* and *E. coli* *MRSA*, *S. aureus* and *E. coli* *S. aureus*	MC3T3-E1 No report MC3T3-E1 MG63 MC3T3-E1	[91] [92] [93] [94] [95]
